# Hunting for the high‐affinity state of G‐protein‐coupled receptors with agonist tracers: Theoretical and practical considerations for positron emission tomography imaging

**DOI:** 10.1002/med.21552

**Published:** 2018-11-18

**Authors:** Vladimir Shalgunov, Aren van Waarde, Jan Booij, Martin C. Michel, Rudi A. J. O. Dierckx, Philip H. Elsinga

**Affiliations:** ^1^ Department of Nuclear Medicine and Molecular Imaging University Medical Center Groningen, University of Groningen Groningen The Netherlands; ^2^ Department of Radiology and Nuclear Medicine Amsterdam University Medical Centers, Academic Medical Center, University of Amsterdam Amsterdam The Netherlands; ^3^ Department of Pharmacology Johannes Gutenberg University Mainz Germany; ^4^ Department of Nuclear Medicine Ghent University, University Hospital Ghent Belgium

**Keywords:** agonist high‐affinity state, experimental design, G‐protein‐coupled receptors, human brain, neurotransmitters, positron emission tomography

## Abstract

The concept of the high‐affinity state postulates that a certain subset of G‐protein‐coupled receptors is primarily responsible for receptor signaling in the living brain. Assessing the abundance of this subset is thus potentially highly relevant for studies concerning the responses of neurotransmission to pharmacological or physiological stimuli and the dysregulation of neurotransmission in neurological or psychiatric disorders. The high‐affinity state is preferentially recognized by agonists in vitro. For this reason, agonist tracers have been developed as tools for the noninvasive imaging of the high‐affinity state with positron emission tomography (PET). This review provides an overview of agonist tracers that have been developed for PET imaging of the brain, and the experimental paradigms that have been developed for the estimation of the relative abundance of receptors configured in the high‐affinity state. Agonist tracers appear to be more sensitive to endogenous neurotransmitter challenge than antagonists, as was originally expected. However, other expectations regarding agonist tracers have not been fulfilled. Potential reasons for difficulties in detecting the high‐affinity state in vivo are discussed.

## 1 INTRODUCTION

Noninvasive imaging of neurotransmitter receptors with positron emission tomography (PET) provides insights into the number of receptors expressed in the brain and the functioning of brain networks. Analysis of the imaging data yields information about the role of particular neurotransmitters in the functioning of the brain in health as well as in neuropsychiatric disorders, including syndromes characterized by cognitive dysfunction.

Neurotransmitters bind to receptors that are either ligand‐gated ion channels (LGICs) or G‐protein‐coupled receptors (GPCRs). For some neurotransmitters, all receptors belong to a single receptor superfamily, for example, all known dopamine receptors are GPCRs. Other neurotransmitters bind to receptors from both superfamilies, for example, there are LGIC‐ and GPCR‐type receptors of the neurotransmitters acetylcholine and glutamate.

**Figure 1:**
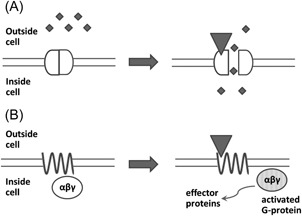
Simplified signaling mechanisms of ligand‐gated ion channels (A) and G‐protein‐coupled receptors (B). If an agonist (represented by a triangle) binds to a channel, the channel opens and ions (represented by small diamonds) can enter the cell. If the agonist binds to a receptor, the G‐protein (represented by an ellipse) dissociates from the receptor complex and activates specific effector proteins

The signaling mechanism of LGICs is comparatively simple and quick—neurotransmitter binding opens the ionic channel that the receptor itself forms. A quick (millisecond time scale) and usually short‐lasting postsynaptic response is thus obtained. GPCRs, on the other hand, affect their downstream signaling pathways through the mediation of trimeric proteins called G‐proteins (Figure 1). The GPCR signaling mechanism is slower (second time scale) and more energy‐consuming than that of LGICs, but long lasting and much more versatile. Due to this versatility, GPCRs are the most popular targets for drugs in clinical use.^1^, ^2^ GPCR‐target drugs or ligands, can be described as agonists or antagonists depending on their effect on the receptor. Agonist drugs can be broadly defined as substances that act like the endogenous neurotransmitter (by binding to the receptor and inducing a physiological response), whereas antagonist drugs bind but do not induce a response and can block the action of the endogenous neurotransmitter.

In vitro studies in membrane homogenates from cultured cells or isolated tissues have shown that in a single population of GPCRs to which antagonist drugs have a single affinity, agonist drugs recognize two distinct receptor subpopulations: one for which they have high affinity and one for which they have low affinity. The existence of a receptor subpopulation that possesses high‐affinity toward the agonists (dubbed “high‐affinity state,” R_high_) has been demonstrated for numerous neurotransmitter GPCRs including dopaminergic,^3^, ^4^ serotonergic,^5^, ^6^, ^7^ muscarinic,^8^ and opioid receptors.^9^ The high‐affinity state is commonly thought to be composed of receptor molecules bound to G‐proteins. A crystal structure of such an activated state of the receptor, in complex with the G‐protein, was first obtained in 2011.^10^

The relationship between G‐protein coupling and high‐affinity toward the agonist gave rise to a hypothesis that the relative abundance of R_high_ may characterize the responsiveness of the synaptic signaling machinery to agonist levels. Indeed, alterations of the fraction of receptors configured in the high‐affinity state, as measured in membrane homogenates, were found in pathological states associated with dysregulation of neurotransmission. For instance, the relative abundance of the high‐affinity state of µ‐opioid receptors was decreased in guinea pigs after chronic morphine treatment,^11^ while the high‐affinity state of muscarinic M_1_ receptors was downregulated in Alzheimer's disease.^12^, ^13^ Upregulation of the high‐affinity state of dopamine D_2_ receptors has been reported in several animal models of psychosis.^14^, ^15^

Assessing the availability of R_high_ may, therefore, provide more valuable information about the state of neurotransmission in vivo than assessing the availability of total receptors. Given that agonists preferentially bind to R_high_, this hypothesis spurred the development of agonist PET tracers and their use for neuroreceptor imaging.

In this study we will review and discuss the molecular basis of the high‐affinity state, inherent advantages and shortcomings of agonist PET tracers stemming from their preferential binding to the high‐affinity state, agonist PET tracers currently available for receptor imaging, experimental methods used for the imaging of high‐affinity state in vivo, and evidence collected with these methods.

## 2 NATURE OF THE HIGH‐AFFINITY STATE OF GPCRS

### 2.1 G‐protein‐dependent high‐affinity state

The canonical view of the nature of the high‐affinity state is based on the so‐called ternary complex model of G‐protein signaling which originates from the studies of agonist binding to β‐adrenergic receptors in membrane homogenates.^16^, ^17^ This model claims that a “ternary” complex must form to launch the G‐protein signaling cascade, consisting of agonist, receptor, and G‐protein. Positive cooperativity between receptor‐agonist and receptor‐G‐protein binding creates the separation of the total receptor population into high‐ and low‐affinity states. For the agonist, receptors complexed with G‐proteins form the high‐affinity state, whereas free receptor molecules represent the low‐affinity state. Indeed, the preference of ligands for the G‐protein‐bound high‐affinity state was found to correlate with their intrinsic activity.^18^, ^19^, ^20^

Several newer and more sophisticated versions of the ternary complex model have been developed to account for pharmacological phenomena such as constitutive activity (presence of baseline signaling in the absence of agonists) and inverse agonism (existence of ligands that decrease rather than increase the level of signaling relative to baseline). These models imply the existence of more than two receptor species with different affinities for the agonist but the main premise remains the same: G‐protein binding is the main factor that determines the receptor's affinity toward the agonist.^21^, ^22^

**Figure 2:**
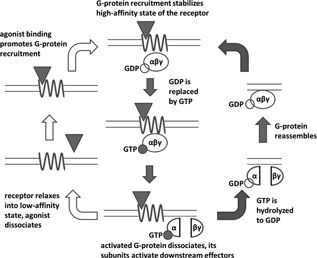
GPCR activation (left circuit, open arrows) and GTP cycle (right circuit, solid arrows). As in Figure 1, the agonist is represented by a triangle, the G‐protein by an ellipse and the receptor by a sinusoid line. The center of the figure shows the “ternary complex” consisting of agonist, receptor and G‐protein. GPCR, G‐protein‐coupled receptor; GTP, guanosine triphosphate

An important feature of the G‐protein‐dependent high‐affinity state is its sensitivity to guanosine triphosphate (GTP). Indeed, the high‐affinity state of GPCRs detected in membrane homogenates usually disappears upon GTP addition.^3^, ^4^, ^5^, ^6^, ^7^, ^8^, ^9^ The reason for this is that the canonical G‐protein signaling cascade involves a so‐called GTP cycle (Figure 2). G‐proteins are heterotrimers and one of their subunits, Gα, has a binding site for guanosine nucleotide (this gives G‐proteins their name). In an inactive G‐protein, this site is occupied by guanosine diphosphate (GDP). Upon G‐protein activation by an agonist‐bound receptor, GDP is replaced by GTP from the cytoplasm, which leads to the dissociation of Gα‐subunit from the Gβ and Gγ subunits (together referred to as Gβγ). The G‐protein splits into two parts, which then activate downstream effectors. If the G‐protein uncouples from the receptor, the receptor quickly returns to its inactive “low‐affinity” state.^23^ Eventual hydrolysis of GTP to GDP in the Gα subunit lets the G‐protein reassemble and bind to the receptor again, which closes the cycle.^24^

Therefore, the GTP cycle acts as a negative‐feedback loop, promoting G‐protein decoupling from the receptors and their (temporary) conversion into the low‐affinity state after agonist binding. Excess GTP shifts the equilibrium toward complete dissociation of G‐proteins from the receptors.

### 2.2 Oligomerization‐dependent high‐affinity state

**Figure 3:**
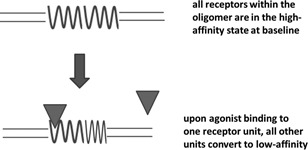
Oligomerization‐dependent high‐affinity state. In this schematic representation, the receptors are drawn as homodimers. Most higher order G‐protein‐coupled receptor complexes are homodimers, heterodimers or tetramers consisting of two different homodimers. The high‐affinity state of the receptor is pictured as a sinusoid, the low‐affinity state as a compressed sinusoid, and the agonist as a triangle is pictured as a compressed sinusoid and the agonist as a triangle

The growing amount of evidence on GPCR oligomerization in cultured cells and living tissues 25 and on the pharmacological relevance of such oligomerization (see Ferre et al^26^ for review) has given rise to the concept of oligomerization‐dependent high‐affinity state. When the agonist interacts with a receptor oligomer, occupying and activating a single receptor unit within it, conformational changes in this receptor influence the conformation of other receptors within the same oligomer and decrease their affinity for other agonist molecules (Figure 3). In other words, separation into high‐ and low‐affinity states is caused by negative cooperativity effects of the agonist binding to oligomerized receptors.^27^

Receptor oligomerization is arguably mainly relevant for the explanation of the interplay between signaling pathways of different receptors^26^: interaction between oligomer subunits is conceptually simpler than interference of downstream cascades. However, among data from radioligand binding studies there are also some results that could be explained better by oligomerization than by G‐protein coupling, such as: (i) GTP‐insensitive high‐affinity agonist binding to dopamine D_3_ and serotonin 5‐HT_2A_ receptors,^28^, ^29^ (ii) detection of high‐ and low‐affinity states of adenosine A_2A_ receptors by antagonist ligands, 30 and (iii) detection of several (more than two) binding sites with different affinities to agonists in the muscarinic M_2_ receptor population.^31^

If there is cooperativity between receptor‐agonist and receptor‐receptor interaction, agonist binding might influence the degree of receptor oligomerization. Some studies indeed report such phenomena^32^, ^33^ but, in general, experimental data on the relationship between ligand binding and oligomerization are contradictory both in terms of whether ligand binding really promotes formation or dissociation of oligomers and whether this action is correlated with intrinsic activity (see Cottet et al^34^, ^35^ for review).

### 2.3 Influence of agonist binding on the high‐affinity state

In both G‐protein coupling and oligomerization‐dependent models of high‐affinity state, agonist binding to the receptor influences receptor interaction with other molecules and thus can alter the relative abundance of the high‐affinity state.

#### 2.3.1 G‐protein‐dependent high‐affinity state

Under conditions where no feedback loops are present, as is the case with in vitro binding studies with nonliving material like membrane homogenates and tissue slices, the relationship between agonist concentration and percentage of receptors in the high‐affinity state at equilibrium is straightforward. In the absence of GTP, agonist binding can only increase G‐protein recruitment. Therefore, increasing agonist concentration will make the percentage of receptors in the “G‐protein‐dependent” high‐affinity state grow from some “floor” value (see Section 2.5) to the “ceiling” value determined by receptor‐G‐protein stoichiometry in the system (100% if the number of available G‐proteins is greater than or equal to the number of receptors). On the other hand, in the presence of excess GTP and negligible GTP hydrolysis, all G‐proteins activated by agonist‐bound receptors will be dissociated and uncoupled from the receptors, so at any agonist concentration, there will be no discernible high‐affinity state.

In living cells and tissues, however, the GTP cycle plays the role of a negative‐feedback loop, which counteracts excess high‐to‐low or low‐to‐high conversion of affinity states caused by the agonist. Depending on the combination of concentrations and kinetic rates, either G‐protein‐recruiting or G‐protein‐dissociating effects of an agonist can become dominant. Indeed, mathematical simulations of GPCR signaling have demonstrated the possibility of both agonist‐induced increase and decrease in the relative abundance of the G‐protein‐dependent high‐affinity state.^22^

#### 2.3.2 Oligomerization‐dependent high‐affinity state

Negative cooperativity in agonist binding to oligomerized receptors implies that increasing agonist concentration will bring more and more receptors into “low‐affinity state.” The percentage of receptors in the high‐affinity state, equal to 100% in the absence of agonist, will decrease to 100%/*N* (*N* is the average number of receptors per oligomer) when the agonist occupies one receptor unit in each oligomer, converting all the other units to low‐affinity state. When agonist concentration raises so high that agonists start to occupy receptors in the low‐affinity state, the relative abundance of the high‐affinity state will fall even lower. There are no well described and widely accepted feedback loops for the oligomerization‐dependent model of the high‐affinity state.

### 2.4 Agonist‐induced receptor internalization

Activation of GPCRs by agonists promotes not only G‐protein binding to them but also their phosphorylation by G‐protein‐coupled receptor kinase (GRK) and internalization mediated by β‐arrestins.^36^ This provides an extra pathway through which the agonist can influence the relative abundance of the high‐affinity state. Internalized receptors are decoupled from G‐proteins (coupled to β‐arrestins instead) and removed from the cell surface to intracellular compartments, where the ionic environment and pH value can be different from extracellular conditions. This makes internalized receptors less accessible (especially for hydrophilic radioligands) and possibly also alters their affinity toward their ligands.

In vitro, β‐arrestin recruitment can happen within minutes.^37^, ^38^ Internalization of dopamine D_2/3_ receptors was observed within the same time frame in vivo and was shown to be dose‐dependent.^39^ Although it is not yet clear whether internalization mainly happens to receptors in the low‐ or high‐affinity state,^40^, ^41^ internalized dopamine D_2/3_ receptors on intact cells and µ‐opioid receptors incubated in a buffer imitating endosomal medium were shown to have decreased affinities toward their ligands.^42^, ^43^

Therefore, high concentrations of an agonist can promote receptor internalization and change the number and relative abundances of receptor subpopulations with different affinities toward imaging radioligands. On the other hand, in internalization‐deficient β‐arrestin knockout mice, baseline binding of dopamine D_2/3_ agonist and antagonist tracers was the same as in wild‐type controls.^44^ This may mean that at basal neurotransmitter levels there already is an equilibrium between neurotransmitter‐induced receptor internalization and recycling.

### 2.5 Relative abundance of high‐affinity state in the absence of the agonist

The oligomerization‐dependent model of the high‐affinity state implies that this is the state in which all receptors are configured in the absence of the agonist.

**Figure 4:**
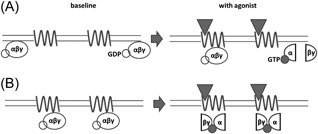
Two extreme modes of receptor‐G‐protein interaction. The agonist is represented by a triangle, the receptor by a sinusoid line and the G‐protein by an ellipse. A, In the collision coupling model, G‐proteins do not stably interact with receptors but agonist action on the receptor promotes G‐protein recruitment to and activation by the receptors, which results in the dissociation of G‐proteins. B, In the precoupling model, G‐proteins are stably bound to the receptors and rearrange their structures upon activation instead of dissociating. GDP, guanosine diphosphate; GTP, guanosine triphosphate

For the G‐protein‐dependent high‐affinity state, its baseline relative abundance, that is, the degree to which G‐proteins interact with the receptors in the absence of agonist is a matter of debate.^45^, ^46^ One extreme view, called collision coupling (Figure 4A), states that in living cells G‐proteins are not normally bound to the receptors but instead interact with them transiently when receptors become activated.^47^ Another extreme view (Figure 4B) states that G‐proteins are always bound (precoupled) to the receptors and do not decouple even after activation, which happens through structural rearrangement of the G‐protein rather than through dissociation.^48^, ^49^, ^50^

On the one hand, collision coupling provides a straightforward interpretation of differences in intrinsic activities of the agonists: agonist efficacy is related to the number of different G‐proteins that an agonist‐bound receptor can bind and activate per unit of time. Decoupling of G‐proteins from the receptors upon activation explains the disappearance of the high‐affinity state upon GTP addition in membrane homogenates. On the other hand, receptors and G‐proteins are known to be coisolated by immunoprecipitation and bioluminescence resonance energy transfer/fluorescence resonance energy transfer (BRET/FRET) experiments with mutated proteins incorporating fluorescent or bioluminescent probe demonstrate close contact between receptors and G‐proteins in the absence of agonists.^45^ Moreover, in BRET studies with α_2_ adrenergic and δ‐opioid receptors, these receptors were found to interact with G‐proteins both before and after activation by agonist.^49^, ^50^

A middle ground between the extreme views is, of course, possible, where some G‐proteins are bound to receptors at baseline but decoupled upon activation, or where G‐proteins are uncoupled at baseline but become bound to receptors upon activation. Moreover, BRET and FRET experiments image the whole population of the receptors, so constant presence of a RET signal, while showing that a fraction of receptors are engaged with G‐proteins, does not exclude the possibility of a rapid turnover of G‐proteins with which these receptors interact.

### 2.6 Summary

The existence of high‐ and low‐affinity states of GPCRs is commonly thought to be due to receptor interaction with G‐proteins. Being a part of the canonical GPCR signaling cascade, the receptor‐G‐protein coupling is directly related to the pharmacological activity of the agonists.

GPCR oligomerization (both homo and hetero), with negative cooperativity in agonist binding within the oligomer, can be an alternative mechanism leading to the formation of receptor subpopulations with different affinities for the agonist. It is plausible that at least for some GPCRs, oligomerization can contribute to the splitting of receptors into high‐ and low‐affinity states instead of, or in addition to, G‐protein coupling.

Both models of high‐affinity state imply that agonists preferentially bind to receptors that are almost ready to launch the signaling cascade, although in the oligomerization model it is so just because agonist binding makes unoccupied receptors “less ready.” Moreover, agonist binding can influence the relative abundance of the high‐affinity state, potentially promoting its formation or disintegration and launching receptor internalization in intact cells and living tissues. Such influence is most directly demonstrated for the G‐protein‐dependent model of the high‐affinity state.

## 3 EXPECTED ADVANTAGES AND DISADVANTAGES OF AGONIST TRACERS RELATIVE TO ANTAGONIST TRACERS

From the notion that agonists preferentially bind to a high‐affinity functional subset of receptors one can logically infer a number of applications in which agonist tracers should be superior, in theory, to antagonist tracers. Note that proposed advantages of agonist tracers mentioned below hold independently of whether the high‐affinity state is G‐protein‐dependent or oligomerization‐dependent.

### 3.1 Applications where agonist tracers have comparative advantage over antagonist tracers

#### 3.1.1 Measurement of synaptic neurotransmission

**Figure 5:**
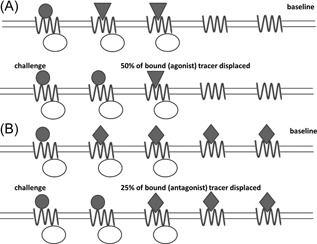
Greater sensitivity of agonist tracers to displacement (“challenge”) by neurotransmitter. Agonist tracers primarily bind to the receptors configured in the high‐affinity state (ie, coupled to G‐proteins), as do neurotransmitters. Therefore, the same change in receptor occupancy by the neurotransmitter displaces a greater fraction of bound agonist tracer (A) than of bound antagonist tracer (B). In this schematic diagram, the endogenous neurotransmitter is pictured as a circle, the agonist ligand as a triangle, the antagonist ligand as a diamond, the G‐protein as an ellipse, and the receptor as a sinusoid line

An endogenous neurotransmitter is an agonist by definition, so it competes with the agonist tracer for the same subset of receptors—receptors configured in high‐affinity state—while an antagonist tracer also binds to receptors in the low‐affinity state that are “ignored” by the neurotransmitter except at very high concentrations. This means that a change in the concentration of neurotransmitter of a given magnitude will lead to greater change in agonist tracer binding compared with antagonist tracer binding (Figure 5).

For some receptor families (eg, serotonin 5‐HT_1A_ and 5‐HT_2A_ receptors), all available antagonist tracers appear to be insensitive to alterations of endogenous neurotransmitter levels.^51^ As agonist tracers are supposed to be more sensitive than antagonist tracers to endogenous neurotransmitter competition, developing agonist ligands is considered a promising way to obtain a tool for the measurement of synaptic neurotransmission via these receptors.^52^

#### 3.1.2 Studies of (pathological) alterations in receptor availability

In Section 1, a few examples were given of how alterations of the percentage of receptors configured in the high‐affinity state can accompany the disease. Since the high‐affinity state is the active form of the receptor involved in signaling and may be primarily affected by the disease, the abundance of the high‐affinity state could be a more meaningful biomarker than the total receptor density. Agonist tracers should then be a convenient tool for pinpointing alterations of the availability of receptors configured in the high‐affinity state in disease.

The results of some in vitro experiments with agonist and antagonist radioligands have supported the hypothesis that agonist tracers are superior to antagonists in detecting pathological changes in neuroreceptor availability. In vitro binding of the 5‐HT_1A_ agonists [^18^F]F15599 and [^18^F]F13640 but not of the antagonist [^18^F]MPPF, in postmortem brain sections of Alzheimer's patients was decreased compared with control brains.^53^, ^54^ In unilateral 6‐hydroxydopamine‐induced lesions of the rat brain (exhibiting dopaminergic neurodegeneration similar to Parkinson's disease in humans, where upregulation of R_high_ is hypothesized), the ex vivo binding of dopamine D_2/3_ agonist [^3^H]NPA was changed to a greater extent than the in vitro binding of D_2/3_ antagonist [^3^H]raclopride.^55^

#### 3.1.3 Measurement of agonist drug occupancy

Many drugs owe their effect to their agonist activity at one or more kinds of receptors. For instance, many antiparkinsonian drugs are D_2/3_ agonists^56^; muscarinic receptor agonists like milameline were tried as treatment of Alzheimer's disease^57^; the mechanism of action of antipsychotics may include not only D_2/3_ antagonism but also 5‐HT_1A_‐agonism^58^, ^59^; the active metabolite of clozapine (also an atypical antipsychotic) acts as an agonist at muscarinic M_1_ receptors^60^; opiate agonists are widely used as analgesics or antitussives and for treating diarrhea and opiate abuse.^61^

Increased sensitivity of agonist tracers to displacement by agonist drugs may be an advantage in occupancy studies: the opioid receptor antagonist [^11^C]diprenorphine failed to detect receptor occupancy by clinically relevant doses of opioid agonists.^62^, ^63^ However, no studies have so far been published, where the sensitivity of an agonist and an antagonist opioid receptor tracer with equal subtype‐selectivity to displacement by an agonist drug was compared head‐to‐head.

Agonist tracers can also complement antagonist tracers in the investigations of the affinity‐state preference of new drugs. The sensitivities of agonist and antagonist tracers to the displacement by the drug can be compared: drugs preferring the high‐affinity state will displace the agonist tracer more readily, while drugs not distinguishing between affinity states will show no difference in displacement efficacy. Two studies attempting this approach have been published^64^, ^65^ but both reported equal displacement of agonist and antagonist tracers by the drug, which can be interpreted in two ways: either the tested drugs were ideal antagonists or the hypothesis of greater agonist tracer displacement by agonist drug does not hold.

### 3.2 Intrinsic shortcomings of agonist tracers

Though the preference for the high‐affinity state makes agonist tracers potentially superior to antagonists in certain imaging applications, it also results in a number of specific difficulties associated with the development and use of agonist tracers.

#### 3.2.1 Lower signal‐to‐noise ratios

The signal‐to‐noise ratio of a PET tracer is proportional to the density of receptors the tracer binds to in the brain (*B*_avail_) and to the tracer's affinity toward these receptors (1/*K*_d_). The density of receptors configured in the high‐affinity state (and thus recognized by agonist tracers) is by definition lower than the total receptor density.

Moreover, estimates of agonist affinity toward the high‐affinity state, acquired in membrane homogenates in vitro, may be systematically higher than the actual affinity in vivo. The reason why it may be so is the negative‐feedback between agonist‐receptor and receptor‐G‐protein binding in the GTP cycle (see Section 2.3), which is part of the G‐protein‐dependent model of the high‐affinity state. Indeed, GTP depletion was shown to increase the affinity of agonist but not antagonist ligands to opioid receptors in cultured cells.^66^ Therefore, affinity and nonspecific binding requirements for agonist tracers are stricter than for antagonists.

#### 3.2.2 Greater likelihood of unwanted pharmacological effects

As agonist tracers preferentially bind to the functional subpopulation of the receptors, they may induce significant physiological responses at a rather low dose, which can distort the experimental results and cause discomfort to the patients.

Indeed, staying below the pharmacological dose range is a concern in opioid receptor imaging with agonist tracers.^67^, ^68^, ^69^ It was also reported as a potential concern in serotonin 5‐HT_1A_ receptor imaging with the agonist [^11^C]CUMI‐101,^70^ even though first tests of the same compound in humans showed no adverse effects.^71^ Exceeding the pharmacological threshold is especially easy with tracers with low specific radioactivity and a related high injected mass of the radioligand. The risk of low specific radioactivity is increased when labeling chemistry is complex. For instance, the dopamine D_2/3_ receptor agonist [^11^C](+)PHNO was originally labeled via a four‐step route, resulting in a relatively low specific radioactivity.^72^ As a consequence, a high incidence of nausea (emesis is a typical effect of D_2_ agonism) was reported in patients injected with [^11^C](+)PHNO,^73^ and it was later found that [^11^C](+)PHNO human PET studies had frequently been performed under nontracer conditions.^74^

## 4 EXISTING PET AGONIST TRACERS FOR GPCR IMAGING IN THE CENTRAL NERVOUS SYSTEM

**Figure 6:**
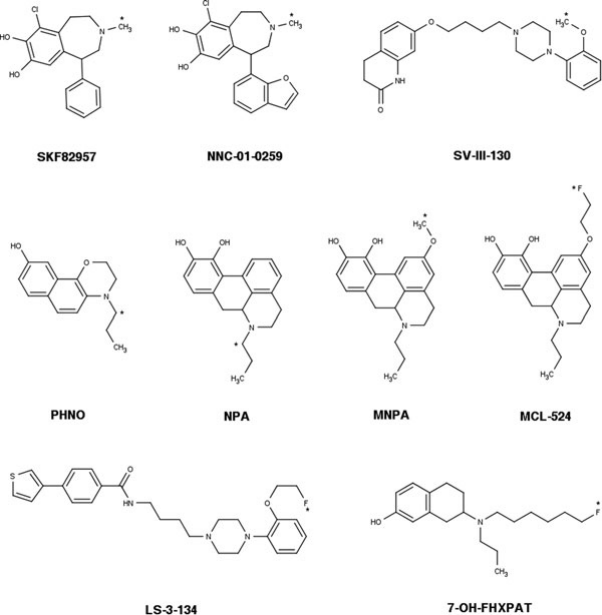
Chemical structures of agonist radioligands for dopaminergic receptors (see also Table 1). The position of the radionuclide in each molecule is indicated by an asterisk

**Figure 7:**
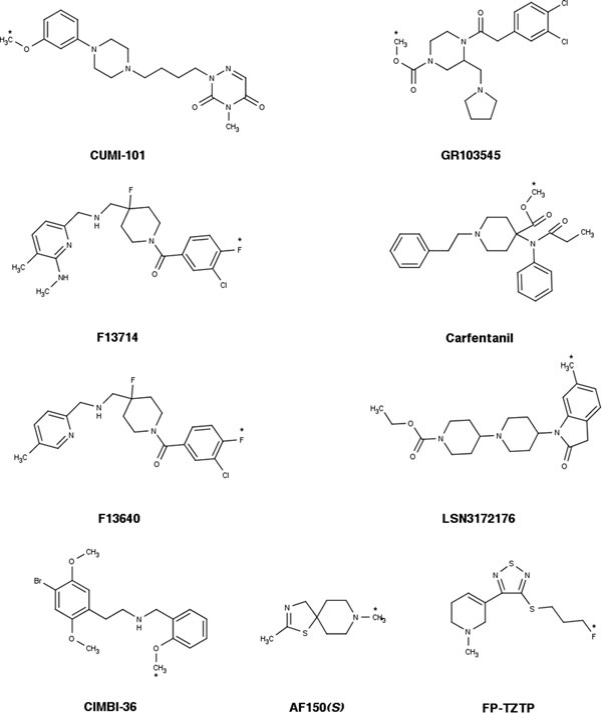
Chemical structures of agonist radioligands for serotonin, opioid, and muscarinic receptors (see also Table 1). [^11^C]PEO is not shown; its structure can be found in Van Waarde et al.^175^ The position of the radionuclide in each molecule is indicated by an asterisk

The greatest number of agonist PET tracers has been developed for the imaging of dopamine D_2/3_ receptors (see 75 for a review). Tracer development efforts in the last two decades have yielded a number of agonist radioligands for other receptors as well. The most promising agonist tracers developed for PET imaging of neuroreceptors are presented in Table 1, Figure 6 and Figure 7.

**Table 1:** Agonist tracers developed for the imaging of high‐affinity state of neuroreceptors

Receptors	Tracer names	In vitro evaluation		In vivo evaluation					Remarks
		Agonism proven by^a^, ^b^	Preference for R_high_ proven by^c^	Rodents	Non‐human primates	Other animals	Humans	Sensitive to endogenous neurotransmitter levels^d^	
Dopamine D_1/5_	(*S*)(+)[^11^C]SKF82957	ND	COMP+ GTPdis+^76^	Rats^77^, ^78^, ^79^	ND	ND	ND	Rats–77	Lipophilic metabolites in brain tissue
	(*S*)[^11^C]NNC 01‐0259	MESS+^80^	ND	ND	81, 82, 83	ND	ND	Primates–80	Lipophilic metabolites in brain tissue
Dopamine D_2/3_	[^11^C]PHNO	ND	GTPdis+^84^ COMP+^85^ SAT−86	Rats^72^, ^87^, ^88^	89	Cats^90^	91 (first)	Rats+^72^, ^87^, ^88^ cats+^90^ primates+^92^ humans+^93^, ^94^	Now primarily used as D_3_‐selective tracer^95^ derivation of ^18^F‐version unsuccessful^96^
	[^11^C]NPA	ND	COMP+^56^, ^85^	Rats^97^	97, 98, 99	Cats^90^ pigs^100^	101 (first)	Cats+^90^ primates+^99^ humans+^102^	Relatively difficult radiosynthesis
	[^11^C]MNPA	ND	COMP+^28^ SAT−86	Mice^44^, ^103^ rats^104^	105, 106, 107, 108, 109, 110	ND	111 (first)	Rats+^104^ mice+^44^ primates+^109^	Lowest BP_ND_ (see Section 5.1.1) among D_2/3_ tracers used in humans
	[^18^F]MCL‐524	ND	COMP+^112^	ND	113	ND	ND	Primates+^113^	Structurally related to NPA and MNPA
Dopamine D_2_	[^11^C]SV‐III‐130	MESS+^114^	ND	ND	114	ND	ND	Primates+^114^	Possible 5‐HT_1A_ binding
Dopamine D_3_	[^18^F]LS‐3‐134	MESS+^115^	COMP−116	ND	115	ND	ND	Primates+^115^	Specific binding seen only after dopamine depletion
	[^18^F]7‐OH‐FHXPAT	ND	GTPdis+^117^	mice, rats^117^	ND	ND	ND	ND	D_3_‐over‐D_2_ selectivity not fully characterized
Serotonin 5‐HT_1A_	[^11^C]CUMI‐101	GTPrec– 118 GTPrec±^119^	ND	ND	70, 120	ND	71, 121	Rodents−122 primates+^123^ humans−124 humans±^125^	Variable intrinsic activity 118, 119, 126 binds to adrenoceptors^118^, ^126^ derivation of ^18^F‐version successful^127^, ^128^
	[^18^F]F13714	GTPrec+ MESS+^129^	GTPdis+^130^	Rats^130^	131	Cats^130^ marmosets^132^	ND	ND	Specific binding is irreversible
	[^18^F]F13640	GTPrec+^133^	ND	Rats^134^	134	Cats^134^	ND	Rats+^134^	Slow, but reversible binding kinetics
Serotonin 5‐HT_2A_	[^11^C]CIMBI‐36	MESS+^135^	ND	Rats, mice (only safety)^136^	137	Pigs^135^	138, 139, 140	Pigs+^141^ primates+^142^ humans−143	Also binds to 5‐HT_2C_ 137 alternative ^11^C‐labeling positions compared 140 derivation of ^18^F‐version unsuccessful^144^
κ‐Opioid	[^11^C]GR103545 also known as (*R*)‐[^11^C]GR89696	PHYS+ (for κ)	COMP±^145^	Mice^146^ (race‐mate)^147^ (eutomer)	68, 69, 148	ND	149, 150	ND	Competition assay shows biphasic binding but this may reflect different affinities for κ
µ‐Opioid	[^11^C]carfentanil (mu‐OR)	PHYS+^151^	ND	Mice^152^ rats^43^	153	ND	153(first)	Rats+^43^ humans+^154^, ^155^, ^156^ humans−157	Derivation of ^18^F‐version successful, no follow‐up^158^
µ/κ‐Opioid	[^11^C]PEO	GTPrec+^159^	ND	Rats^159^	ND	ND	ND	ND	Derivation of ^18^F‐version successful^160^, ^161^
Muscarinic M_1_	[^11^C]LSN3172176	GTPrec+^162^	COMP−162		163, 164	ND			Imperfect subtype‐selectivity
	[^11^C]AF‐150(*S*)	ND	COMP+^20^	Rats^165^, ^166^	ND	ND	ND	Rats±^166^	Low signal‐to‐noise ratios
Muscarinic M_2_	[^18^F]FP‐TZTP	PHYS+^167^	ND	Mice^168^ rats 169, 170, 171	170	ND	172(first)	Primates+^173^	^11^C‐version created, no follow‐up^174^

Abbreviations GDP, guanosine diphosphate; GTP, guanosine triphosphate; ND, no data available.

^a^ Coding of experimental paradigms aiming to confirm agonism: MESS monitoring secondary messenger levels in functional assays in vitro*;* GTPrec monitoring GTP recruitment to G‐proteins in vitro; PHYS monitoring physiological or behavioral effects of the compound in vivo or ex vivo.

^b^ Works confirming functional agonism are cited only if the preference for R_high_ has not been directly confirmed.

^c^ Coding of experimental paradigms aiming to confirm preferential binding to R_high_: COMP obtaining a biphasic competition curve in vitro; SAT obtaining a biphasic saturation curve in vitro; GTPdis detecting the loss of specific binding upon GTP or GppNHp addition in vitro.

^d^ Coding of the outcomes of studies confirming sensitivity to endogenous neurotransmitter levels (and also agonism and R_high_ preference): +, positive outcome; −, negative outcome, ±, ambiguous results.

### 4.1 Definition and properties of an agonist tracer

An agonist tracer is usually defined as “a radiolabeled analog of a ligand with agonist activity.” There are many ways to confirm and measure the degree of agonist activity: behavioral or ex vivo studies examining the physiological effect of the drug, functional in vitro assays measuring the levels of certain secondary messengers, or the recruiting of proteins involved in signaling cascades to the receptors.

Because the intrinsic activity of a ligand is known to be correlated with the ratio of its affinities to the high‐ and low‐affinity receptor states,^18^, ^19^ it seems evident that agonists will preferentially bind to the high‐affinity state. However, agonism does not necessarily imply preferential binding to receptor‐G‐protein complexes, since noncanonical signaling pathways do exist. One example is cariprazine, a drug which was recently labeled with carbon‐11 and evaluated as a dopamine D_2/3_ receptor PET tracer. This compound showed partial D_2/3_ agonist activity in secondary messenger assays but did not recruit G‐proteins in vitro.^176^, ^177^, ^178^ Observations of G‐protein recruitment may also differ between in vitro setups. For instance, [^11^C]CUMI‐101, a tracer for serotonin 5‐HT_1A_ receptors, was defined as an agonist based on the [^35^S]GTPyS assay (indirect measurement of G‐protein recruiting to receptors) in membrane homogenates from cell cultures expressing recombinant human receptors but was later found to act as an antagonist when the same assay was done in primate and rat brain homogenates.^118^, ^119^

Therefore, the most certain proof of the agonist radioligand's in vitro preference to the high‐affinity state is directly demonstrating that it recognizes high‐ and low‐affinity states of its receptor in natural tissue or in transfected cell culture. It is worth noting that for some agonist tracers, preferential in vitro binding to R_high_ was demonstrated only after the tracer had been evaluated in vivo (compare 105 and 28), while for some other radioligands the capability to discern affinity states in vitro was not assessed at all.^80^, ^135^

### 4.2 In vivo evaluation of a PET neuroreceptor tracer

Characteristics desirable for a PET tracer for brain imaging include the ability to pass the blood‐brain‐barrier, a low degree of metabolism, a high contrast between target (specific) and nontarget (nonspecific) binding, and pharmacokinetics that can be reliably quantified from a 60 to 90 minute‐long PET scan (see 179, 180, 181 for review). An important aim in PET imaging is the measurement of synaptic neurotransmission. For this reason, neuroreceptor tracers are tested for the sensitivity of their binding to changes of endogenous neurotransmitter levels, and agonists are supposed to be more sensitive than antagonists.

Neuroreceptor tracers are usually evaluated in rodents or non‐human primates before being moved to human studies. In non‐human primates, one can investigate the binding of the tracers with high spatial detail using clinical PET cameras. Evaluation in rodents is cheaper and enables the use of more invasive methods but interspecies differences in rodent, primate, and human physiology can be a confounding factor. In addition, the small size of rodents forces the researchers to use dedicated nonclinical “micro‐PET” cameras and does not permit to reliably image minor brain structures. To strike the right balance between controllability of the experimental conditions and image quality, tracers are sometimes evaluated in (relatively) large mammals, such as cats or pigs.

### 4.3 Availability of agonist PET neuroreceptor tracers

Agonist PET tracers can be divided into three categories (see Table 1). The first category includes radioligands that have reached the stage of in vivo evaluation in humans. The dopamine D_2/3_ ligands [^11^C](+)PHNO, [^11^C]NPA, and [^11^C]MNPA, the serotonin 5‐HT_1A_ ligand [^11^C]CUMI‐101, the serotonin 5‐HT_2A_ ligand [^11^C]CIMBI‐36, the κ‐opioid ligand [^11^C]GR103545, the µ‐opioid ligand [^11^C]carfentanil, and the muscarinic M_2_ receptor agonist [^18^F]FP‐TZTP fall into this category. The binding of most of these ligands has been shown to be sensitive to changes of endogenous neurotransmitter levels, although for [^11^C]CIMBI‐36 and [^11^C]CUMI‐101 the acquired results have been conflicting, and the sensitivity of [^11^C]GR103545 to endogenous opioids has not yet been evaluated. Many ligands from category one are used in PET imaging for other reasons than their agonist properties. [^11^C](+)PHNO, for example, is frequently selected as a tracer because it binds preferentially (though not selectively) to dopamine D_3_ receptors, and its preference for this subtype is higher than that of dopamine D_2/3_ antagonist ligands like [^11^C]raclopride.^95^ [^11^C]CUMI‐101 is used in clinical studies,^182^, ^183^ not because of its ability to image the high‐affinity state of serotonin 5‐HT_1A_ receptors (which is actually under doubt—see below) but because of its high subtype‐selectivity and imaging contrast. [^11^C]carfentanil and [^18^F]TZTP are the only tracers available for µ‐opioid and muscarinic M_1_ receptors, so in their case, there are no antagonist tracers with which they could be compared head‐to‐head.

The second category contains radioligands that have not (yet) been tested in humans but have been evaluated in non‐human primates. The dopamine D_1/5_ ligand (*S*)[^11^C]NNC 01‐0259, the dopamine D_2/3_ ligand [^18^F]MCL‐524, the dopamine D_2_ ligand [^11^C]SV‐III‐130, the dopamine D_3_ ligand [^18^F]LS‐3‐134, the serotonin 5‐HT_1A_ ligands [^18^F]F13714 and [^18^F]F13640, and the muscarinic M_1_ ligand [^11^C]LSN3172176 belong to this group. The binding of the dopamine D_2_, D_3_, and D_2/3_ ligands from this category has been shown to be sensitive to changes of endogenous neurotransmitter levels but for the dopamine D_1/5_ the results were negative, and there are currently no D_1/5_ agonists or antagonists that would be sensitive to endogenous dopamine levels. Sensitivity of [^18^F]F13714, [^18^F]F13640, and [^11^C]LSN3172176 to endogenous serotonin/acetylcholine has not yet been evaluated in primates, although [^18^F]F13640 was sensitive to serotonin levels in rats. Some of these compounds were radiolabeled with ^18^F or ^18^F‐versions of previously developed ^11^C‐tracers because the longer half‐life of fluorine‐18 makes an imaging agent more suitable for wide‐scale clinical use. [^18^F]sufentanil has been created as a longer half‐life alternative to [^11^C]carfentanil but was not further developed.^184^

The third and last category contains tracers that have only been evaluated in rodents. The dopamine D_1/5_ ligand (*S*)(+)[^11^C]SKF82957, the dopamine D_3_ ligand [^18^F]7‐OH‐FHXPAT, the µ/κ‐opioid agonist [^11^C]PEO, and the muscarinic agonist [^11^C]AF‐150*(S)* fit in this category.

Binding of (*S*)(+)[^11^C]SKF82957 is known to be insensitive to changes of endogenous neurotransmitter levels. Available data for [^11^C]AF‐150(*S*) in this regard are inconclusive, and for other tracers from this category sensitivity to endogenous neurotransmitter levels has not yet been evaluated.

In the process of tracer development, some agonist tracers were found to have issues that hamper their routine application. Two radioligands for dopamine D_1/5_ receptors, (*R*)‐[^11^C]SKF82957 and (*S*)‐[^11^C]N‐methyl‐NNC 01‐0259,^81^, ^185^ are converted to lipophilic radioactive metabolites that penetrate into the brain and can confound the interpretation of imaging results, though this can be remedied by inhibiting the activity of the enzyme catechol‐O‐methyl transferase.^77^ Another D_1/5_ agonist, [^11^C]SKF75670, was originally developed together with [^11^C]SKF82957 but was abandoned because of inferior signal‐to‐noise ratios. Since it is structurally very similar to [^11^C]SKF82957, it may show the same unfavorable in vivo metabolism.^78^ The development of (*S*)‐[^11^C]N‐methyl‐NNC 01‐0259 was halted since tests examining its sensitivity to endogenous dopamine levels yielded a negative result.^80^ The D_3_‐selective agonist tracer [^18^F]LS‐3‐134 was shown to specifically bind to D_3_ receptors in monkey brain but specific binding was only measurable under dopamine depletion conditions.^115^ The muscarinic M_1_ receptor tracer [^11^C]AF‐150(*S*) showed both specific binding and sensitivity to endogenous acetylcholine levels in the rat brain but the low signal‐to‐noise ratios of this ligand cast doubt on its suitability for further research.^165^, ^166^

To demonstrate the preferential binding of agonist tracers to receptor high‐affinity state, a head‐to‐head comparison with reference tracers binding to all receptors (ie, antagonist tracers) is required. For D_1/5_, D_2/3_, 5‐HT_1A_, and 5‐HT_2A_ agonist tracers counterpart antagonist tracers are available, which possess matching pharmacological selectivity (ie, bind with similar relative affinities to the same receptor subtypes within the relevant region of interest as their “corresponding” agonist tracers) and, in case of D_2/3_ and 5‐HT_2A_ ligands, sensitivity to endogenous neurotransmitter levels.^51^, ^186^, ^187^, ^188^ The same is true for the κ‐opioid agonist [^11^C]GR103545, which was developed along with its antagonist counterpart [^11^C]LY2795050,^189^ and for the µ/κ‐opioid agonist [^11^C]PEO, which can be used in conjunction with the µ‐partial agonist and κ‐antagonist [^11^C]buprenorphine.^159^ For the µ‐opioid agonist [^11^C]carfentanil, the M_2_ agonist [^18^F]FP‐TZTP and M_1_ agonists [^18^F]AF‐150(*S*) and [^11^C]LSN3172176 there are no suitable antagonist counterparts.^175^, ^190^ In theory, non‐subtype‐selective radioactive antagonists might be used in head‐to‐head comparisons with radioactive agonists if receptors to which the antagonist, but not the agonist, binds are fully blocked with a nonradioactive drug, but the feasibility of that approach is questionable because of the possible pharmacological effects of such blockade.

In conclusion, agonist tracers for PET imaging of dopamine D_2/3_, serotonin 5‐HT_1A_ and 5‐HT_2A_, µ and κ‐opioid, and M_2_ muscarinic receptors are available. Agonist tracers for dopamine D_1/5_ and muscarinic M_1_ receptors have issues that make their use for PET imaging problematic. Preference for the high‐affinity state in vitro has only been directly demonstrated for D_1/5_ and D_2/3_ tracers, some 5‐HT_1A_ tracers and the M_1_ tracer [^18^F]AF‐150(*S*). Agonist tracers for D_2/3_, 5‐HT_1A_, 5‐HT_2A_, and κ‐opioid receptors can be matched with antagonist tracers binding to the same receptors for head‐to‐head comparisons.

## 5 PROVING THE EXISTENCE OF THE HIGH‐AFFINITY STATE IN VIVO

### 5.1 Outcome measures of in vivo experiments

In vivo experiments with neuroreceptor radioligands measure the availability of the receptors and changes in this availability in response to alterations of endogenous neurotransmitter levels or administration of exogenous drugs. Binding potentials (BPs) and target/nontarget ratios are “raw” measures representing receptor availability, which can later be recalculated to receptor densities or occupancies.

#### 5.1.1 Binding potentials

The typical outcome measure of in vivo imaging experiments is the BP. This parameter is defined as the product of the density of binding sites (*B*_max_) and the affinity of the radioligand for these sites (inverse of the dissociation constant, 1/*K*_d_). BP is equal to the ratio of the concentrations of specifically bound and free ligand in the tissue of interest at equilibrium, provided that the administered dose of radioligand is sufficiently low (see the Appendix for more explanation). BP is estimated by fitting a kinetic model to measured time‐activity curves. Time‐dependent radioactivity in the region of interest and a reference region in the brain can be measured by PET imaging, and plasma radioactivity can be determined by blood sampling.^191^ Note that in vivo not all receptors may be available for binding, as they can be internalized, converted to low‐affinity state (for an agonist) or occupied by neurotransmitter, so in the in vivo context the term *B*_avail_ is more suitable than *B*_max_.

Given the difficulty of determining the true concentration of the free ligand in the living tissue, other concentrations proportional to free ligand concentration in tissue are substituted in its place. Specifically, bound concentration is related to free plasma concentration (BP_F_), total plasma concentration (BP_P_), or “nondisplaceable” concentration (BP_ND_), that is, the total concentration of free and nonspecifically bound ligand in the tissue.^192^ It is reasonable to assume that free ligand concentrations in the plasma and in the interstitial liquid of the brain tissue are equal at equilibrium, so BP_F_ can be considered the “true” BP.

#### 5.1.2 Target/nontarget ratios

When regions of interest are small relative to PET camera resolution (ie, subsections of rodent brain) it is often hard to obtain a reliable time‐activity curve with high temporal resolution. Also, in situations when a lot of experimental conditions have to be tested and compared, it is often infeasible to obtain time‐activity curves by PET or to sacrifice large groups of animals at different time points. In these situations, one can take advantage of the “pseudoequilibrium” state when the concentration ratios between receptor‐rich and receptor‐free tissues remain constant even as absolute concentrations are changing. For a typical neuroreceptor ligand, one can reasonably expect the pseudoequilibrium state to be reached within half an hour after injection. Once the time range in which the pseudoequilibrium exists is validated, specific binding can be estimated from tissue concentrations at a single time point within this time range. Such concentrations can be obtained by ex vivo dissection and radioactivity counting or from a static PET scan. Target/nontarget concentration ratios can be used as is or be recalculated to specific binding ratios (SBRs):

$$\text{SBR}\;=\;\;\frac{T-\text{NT}}{\text{NT}}\;=\;\frac{T}{\text{NT}}-1.$$

where T and NT are radioligand concentrations in receptor‐rich (“target”) and receptor‐poor (“nontarget”) regions of interest. In the absence of specific binding, SBR = 0, while T/NT = 1.

“Raw” specific binding, that is, the difference between radioligand concentrations in receptor‐poor and receptor‐rich regions can also be used as an outcome measure. However, when specific ligand binding is not normalized to the nonspecific binding at the same time point, its value is prone to intrasubject variations in pharmacokinetics. Therefore, the use of binding ratios is preferred.

#### 5.1.3 Available receptor density

Available receptor density (*B*_avail_) can be estimated in a saturation experiment. Specific binding of a radioligand is determined by two parameters: the density of binding sites in the region of interest (*B*_avail_) and the affinity of the radioligand toward these receptors (1/*K*_d_). To independently estimate these two parameters, bound and free radioligand concentrations at equilibrium have to be estimated at least at two different injected doses (for radiotracers, injected dose is usually varied by changing molar radioactivity). Bound concentration can be estimated from a difference in equilibrium tracer concentrations between the target and nontarget regions, while free concentration can be back‐calculated from the binding potential (see Appendix). *B*_avail_ can then be quantified by regression analysis. In PET imaging, the regression is often performed on “linearized” binding data: binding potential is plotted against absolute specific binding (Scatchard plot). Scatchard plot requires a simple linear regression and is therefore straightforward but also bias‐prone, as the *X* and *Y* axes are not independent of each other. Alternatively, *B*_avail_ can be estimated by nonlinear regression of the binding curve (bound vs free concentration plot). For in vitro assays, where free and bound ligand concentrations can be independently estimated, nonlinear regression is the gold standard. However, for in vivo experiments the Scatchard plot has remained popular.

#### 5.1.4 Receptor occupancy

Displacement of a tracer from its receptors by a competing ligand decreases the binding potential of the tracer. Receptor occupancy can be calculated as the change in binding potential or target‐nontarget ratio after drug administration, relative to baseline. The same holds for the occupancy of the receptors by endogenous neurotransmitter, when drugs stimulating neurotransmitter release or depletion are administered.

### 5.2 Experimental paradigms used to demonstrate the existence of high‐affinity state in vivo

To demonstrate that agonist tracers preferentially bind to a certain “high‐affinity” subset of receptors in vivo three approaches have been used (summarized in Table 2). One approach is to directly measure the available binding site densities for agonist and antagonist tracers and demonstrate that the binding site density available to the agonist is lower. Another is to infer the ratio of high‐affinity binding site density to total binding site density from the results of experiments where tracers compete for binding to the receptors with unlabeled ligands. A third approach is to demonstrate that agonist, but not antagonist, binding can be influenced by manipulations of receptor‐G‐protein coupling.

**Table 2:** Experimental paradigms used for the detection of high‐affinity state in vivo

Approach	Experimental paradigm	Minimum ligand set necessary	Minimum number of experimental conditions	Results confirming the presence of high‐affinity state In vivo	Shortcomings		Examples
					Risk of pharmacological effects	Other	
Binding site density comparison	Saturation experiment	Labeled antagonist + la ‐ beled agonist	4 Minimum 2 dose levels per radioligand	Lower *B*_max_ value for agonist tracer than for antagonist tracer	Yes		90, 122, 193
	*B*_avail_ = BP_F_ × *K*_d_	Labeled antagonist + labeled agonist	2 Single dose level per radioligand	Lower *B*_max_ value for agonist tracer than for antagonist tracer	No	Requires arterial input with free fraction in plasma, in vitro *K*_d_ is likely not equal to in vivo *K*_d_	70
	Correlation analysis	Labeled antagonist + labeled agonist	2 Single dose level per radioligand	Presence of the main trend with upward or downward deviations from it for certain regions of interest	No	See Section 5.2.1, third subsection	70, 132, 137, 139
	Imaging in disorders	Labeled antagonist + labeled agonist	4 Single dose level per radioligand, imaging in healthy and diseased state	Same specific binding for antagonist tracer in control and diseased state, different specific binding for agonist tracer	No	Upregulation or downregulation of high‐affinity state has to be demonstrated in vitro	103, 194, 195
Competition studies	In vivo displacement curves	Labeled antagonist + unlabeled agonist	5 Minimum number of dose levels to distinguish between mono‐ and biphasic curves	Displacement curve better explained by biphasic than by monophasic model	Yes	large number of dose levels to test	86, 196, 197, 198
	Neurotransmitter challenge	Labeled antagonist + labeled agonist + stimulator of neurotransmitter release or depletion	4 Single dose level per radioligand, imaging before and after challenge	Greater displacement of agonist tracer by the challenge of same magnitude	Yes	see Section 5.2.2	90, 98, 106
	Exogenous drug challenge	Labeled antagonist + labeled agonist + unlabeled agonist	4 Single dose level per radioligand, imaging before and after challenge	Greater displacement of agonist tracer by the challenge of same magnitude	Yes		86, 107, 196, 199, 200
Probing the nature of the high‐affinity state	Sensitivity to G‐protein coupling	Labeled antagonist + la ‐ beled agonist + agent for G‐protein decoupling	4 Single dose level per radioligand, imaging before and after G‐protein decoupling	G‐protein decoupling decreases specific binding for the agonist but not for the antagonist radioligand	No	Requires intrathecal or intracerebral injections, does not look at what fraction of total receptors are in the high‐affinity state	Proof of concept presented in^15^

It is important to emphasize that to compare agonist and antagonist tracers with each other their pharmacological selectivity profiles (ie, relative binding affinities toward different receptor subtypes) should be identical within the region of interest used for comparison. Otherwise, any detected difference in binding behavior could be attributed to the relative preference of one of the tracers toward a certain receptor subtype.

In principle, all experiments described below can be performed not only with PET tracers labeled with short‐lived positron‐emitting isotopes but also with radioligands labeled with long‐lived isotopes such as tritium (^3^H). On the one hand, this makes the experimental paradigm nontranslatable to the clinical setup. On the other hand, this enables the use of elegant approaches like the double‐tracer study, where radioligands labeled with short‐lived (eg, ^11^C) and long‐lived (eg, ^3^H) isotopes are compared head‐to‐head in the same group of animals.^194^, ^196^

#### 5.2.1 Approach 1: comparing binding site densities

##### Saturation study

As explained above, a minimum of two different radioligand doses needs to be tested to estimate binding site density (*B*_avail_). More doses will add precision and can reveal potential cooperativity effects or the presence of multiple binding sites with different affinities (eg, receptor affinity states), provided that radioligand binding to all these sites is distinguishable from nonspecific binding. However, published PET studies comparing binding site densities of agonist and antagonist tracers were restricted to two doses.^90^, ^193^ Another study used single time point SBRs as outcome measure and built saturation curves based on 9 to 10 data points.^122^

In two‐dose PET studies aimed at quantifying *B*_avail_, the low dose corresponds to the minimum amount of radioligand that can be injected, that is, the “tracer dose,” which should occupy less than or equal to 10% of the receptor population in the region of interest. The high‐dose is chosen to occupy about two‐thirds of that population.^90^, ^193^

##### Extracting density values from true binding potential measurements

It should be noted that performing a saturation assay with agonist radioligands can lead to unwanted and dangerous pharmacological effects, especially in the case of opioid ligands.^68^, ^69^

In a head‐to‐head comparison of 5‐HT_1A_ agonist and antagonist tracers, Kumar et al^70^ attempted to circumvent this problem by comparing the “true” binding potentials (BP_F_) for the two tracers at low injected dose instead of performing a second high‐dose scan to independently measure *B*_avail_ and *K*_d_. Given that BP_F_ = *B*_avail_/*K*_d_, *B*_avail_ can arguably be calculated from the BP_F_ value using in vitro *K*_d_ value for the corresponding tracer. However, there are two problems with this approach. First, to calculate BP_F_, one needs to obtain an arterial input curve and free fraction in plasma for the investigated radioligand, in addition to the time‐activity curve for the region of interest. Such a large amount of input data makes BP_F_ prone to experimental error and bias. Second, the in vivo *K*_d_ of the radioligand is not necessarily equal to the in vitro *K*_d_, especially if the latter is measured for receptors from a different animal species or in transfected cells.

##### Studying correlation between regional binding of agonist and antagonist

Binding potentials or target/nontarget ratios for an agonist tracer in various brain regions can be plotted against the corresponding measurements for an antagonist tracer, to examine their correlation. Such a plot may provide insight into the relationship between the densities of available binding sites for agonist and antagonist tracers while staying below the “tracer” threshold. If agonist binding in a certain brain region lies above the main trend on the correlation graph, it suggests that the relative abundance of receptors in the high‐affinity state in this region is higher than average, and vice versa.

This approach, however, has many limitations. If the relative abundance of the high‐affinity state is drastically different in each region, the correlation graph will be meaningless: there will be no main trend to pinpoint deviations from. If the relative abundance of the high‐affinity state is the same in all regions, the correlation graph will be a straight line, revealing no differences in agonist and antagonist binding and thus no evidence in favor of the existence of the high‐affinity state. Therefore, analysis of the correlation between agonist and antagonist binding cannot be the sole method of looking for the existence of high‐affinity state but can be an extra piece of data analysis in experiments based on other paradigms.

##### Studying agonist binding in disorders presumably caused by high‐affinity state dysregulation

In vitro experiments in membrane homogenates suggest that some neuropsychiatric disorders are accompanied by alterations in the relative abundance of the high‐affinity state in a given receptor population, while changes in overall receptor density relative to the healthy condition are either absent or much less pronounced. One example is animal models of psychosis where the high‐affinity state of dopamine D_2/3_ receptors is upregulated.^14^, ^201^

Therefore, another way to demonstrate the existence of a high‐affinity state in vivo is to show that its upregulation (or downregulation) can be noninvasively detected by agonist tracers. If the relative abundance of the high‐affinity state is altered but the overall receptor density remains (relatively) constant, the binding of the agonist but not of the antagonist tracer will be different in the diseased state relative to the healthy state. Ratios of BP or SBR values for agonist and antagonist tracers can be used as outcome measures to normalize for possible concomitant alterations in total receptor density.^202^

In this paradigm, the binding of each tracer only has to be assessed at a low and pharmacologically inactive dose. However, one has to demonstrate that the relative abundance of high‐affinity state really differs between healthy and diseased states, using experimental approaches other than PET (typically, in vitro assays). Moreover, in the diseased state, alteration of the relative abundance of the high‐affinity state may be accompanied by alterations in other parameters relevant for radioligand binding. For instance, changes in baseline neurotransmitter levels also differentially affect the binding of agonist and antagonist tracers (agonist binding is changed to a greater extent). Concomitant changes in several parameters pressing agonist tracer binding in different directions can offset each other, leading to little or no change in overall receptor availability to the agonist tracer compared with the healthy state.

#### 5.2.2 Approach 2: studying tracer vulnerability to displacement by an unlabeled competitor

One important difference between tracer‐drug competition experiments in vitro and in vivo is that in the latter case the concentration of both tracer and drug at the receptors is not constant. While for the radioligand a true equilibrium between its concentrations in blood and brain tissue can be achieved by using bolus‐plus‐infusion injection scheme, the same is virtually infeasible for the unlabeled drug (tissue concentrations of which are much harder to monitor). Nevertheless, one can usually safely assume that the pharmacokinetics of the competing drug are dose‐linear within the investigated dose range, so the degree of displacement of the tracer by the drug is also dose‐linear.

##### Building in vivo displacement curves

In vitro, the high‐affinity state is detected by displacing an antagonist radioligand with ever increasing concentrations of unlabeled agonist drug. When the remaining specific binding of the radioligand is plotted against agonist concentrations, the displacement is shown to proceed in two phases: agonist first displaces the radioligand from high‐affinity sites then from low‐affinity sites. The same displacement curve can be built in vivo by plotting binding potentials or target/nontarget ratios for an antagonist tracer against an administered dose of unlabeled agonist drug.

The advantage of this paradigm is that it does not require an agonist radioligand. Antagonist radioligands are much more numerous than agonist radioligands, so the displacement curve paradigm is currently applicable to a wider range of receptors than other paradigms mentioned below.

The downside, however, is that the generation of a displacement curve is a laborious undertaking. The shape of the biphasic curve is determined by five parameters: maximum binding level (at no displacement), minimum binding level (full displacement), agonist affinities for high‐ and low‐affinity states, and the percentage of receptors in the investigated population configured in the high‐affinity state. This means that at least five different dose levels (including zero) have to be used to test whether the obtained curve is monophasic or biphasic.

Therefore, studies that used the displacement curve approach typically used SBRs obtained ex vivo at a single time point from large numbers of rodents,^86^, ^196^ though the use of PET scanning in primates has also been reported.^197^, ^198^ The actual number of dose levels tested was 6 to 9 in rodent and 9 in non‐human primates.

##### Comparing vulnerability to displacement by unlabeled agonist

An agonist tracer should be more vulnerable than an antagonist tracer to displacement (or “challenge”) by other agonists because it competes with them for the same subpopulation of receptors. Displacement can be elicited by administering an appropriate agonist drug or by stimulating endogenous neurotransmitter release.

The advantage of using exogenous agonist drugs for displacement is that these drugs can be selected to be subtype‐specific and to only occupy the receptor population that is being imaged (or even a defined subset of this population if the tracer binds to more than one receptor subtype).

On the other hand, manipulating neurotransmitter levels has the advantage of being “natural”: one looks at the competition of the tracer with the endogenous ligand, the action of which on the receptors is thought to govern the functioning of the brain [see Laruelle^187^ and Finnema^51^ for reviews]. One can also reasonably expect that the competition will only happen at receptors that are really situated in the synapses. Moreover, neurotransmitter levels can be both increased and decreased relative to the baseline. In the latter case, the expected result is greater increase, rather than greater decrease, of binding for the agonist tracer. However, manipulating neurotransmitter release has its downsides, too. First, the effect vs time relationship between the administration of the drug that stimulates a rise or fall in endogenous neurotransmitter level and synaptic receptor occupancy is more complex than when receptors are occupied with exogenous agonist. Second, the released neurotransmitter can act on other receptor subtypes beyond the one being imaged. Third, some drugs used to manipulate neurotransmitter levels are known to manipulate levels of several neurotransmitters at once (eg, amphetamine stimulates both dopamine and norepinephrine release). The lack of selectivity regarding what neurotransmitter is manipulated and which receptors are occupied can confound the interpretation of cause‐and‐effect relationships.

**Figure 8:**
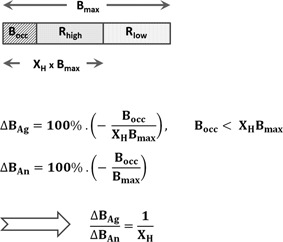
Relationship between agonist and antagonist tracer displacement (∆*B*_Ag_ and ∆*B*_An_) and the fraction of receptors occupied by competing agonist drug or neurotransmitter. *B*_max_ is the total receptor density available at baseline, *X*_H_ is the fraction of receptors configured in the high‐affinity state, *B*_occ_ is the amount of receptors occupied as a result of the challenge. If *B*_occ_ < *X*_H_ *B*_max_, that is, not all high‐affinity state receptors become occupied, the ratio of relative decreases of agonist and antagonist tracer binding is constant and equal to 1/*X*_H_

If the “tracer condition” is satisfied (radioligands occupy a negligible fraction of all receptors), the ratio of agonist and antagonist radioligand vulnerabilities to displacement by a challenge is a constant value as long as less than 100% of the high‐affinity state is occupied as a result of the challenge (Figure 8). Therefore, in theory, a single dose of agonist drug or neurotransmitter level manipulator should provide enough information to compare the vulnerability of agonist and antagonist tracers. In practice, because the actual percentage of receptors in the high‐affinity state is unknown, several doses are often tried, resulting in occupancies of up to 100%,^86^, ^90^, ^107^, ^196^ except in the human studies where the maximum challenge magnitude is limited by ethical considerations.^93^, ^102^

An important limitation of the vulnerability comparison paradigm is that preference for the high‐affinity state is not the only factor influencing the vulnerability of the radioligand to displacement by unlabeled drugs. For instance, many preclinical in vivo tracer binding experiments are performed in anesthetized animals, and isoflurane and ketamine anesthesia were found to increase the baseline binding of agonist D_2/3_ tracers, exaggerating the vulnerability of agonist tracers relative to antagonists.^109^, ^203^ The mechanism of such selective influence is unclear, although there are reports that anesthetics interfere with receptor‐G‐protein (un)coupling 204, 205 and alter endogenous neurotransmitter levels.^206^, ^207^

Furthermore, D_2/3_ antagonist tracers are known to differ between themselves in the sensitivity to changes in dopamine levels.^187^, ^208^ The underlying reasons can be more or less favorable binding kinetics (see Finnema et al^51^, ^209^ for discussion) or differences in affinity toward the surface and internalized receptors.^210^, ^211^ Although a head‐to‐head comparison of D_2/3_ agonist [^11^C]MNPA and D_2/3_ antagonist [^18^F]fallypride in internalization‐deficient mice demonstrated that competition with the neurotransmitter is sufficient to explain short‐term (though not long‐term) changes in binding for both tracers,^44^ this situation can be different for other receptor types.^43^

#### 5.2.3 Studying vulnerability to G‐protein uncoupling in vivo

Addition of GTP or its analogs decreases specific binding of agonist but not antagonist ligands in vitro, so uncoupling of G‐proteins induced in vivo should lead to the same effects.

Seeman^15^ demonstrated that GTP addition to tissues extracted from an animal after D_2/3_ agonist radioligand injection accelerates radioligand dissociation from D_2/3_ receptors in the tissue and proposed the use of pertussis toxin to promote G‐protein decoupling from the receptors in vivo. Indeed, physiological effects of dopamine and opioid receptor agonists were inhibited by pertussis toxin injections.^212^, ^213^, ^214^

This approach probes the nature of the high‐affinity state, that is, it seeks an answer to the question “is G‐protein binding to the receptor significant for agonist binding to the receptor?” However, this question is not the same as “do agonists bind to a subset of all receptors?,” which is addressed in other paradigms. As discussed in Section 2, all receptors may be precoupled to G‐proteins. Moreover, G‐protein decoupling agents (pertussis toxin or anything else) will have to be introduced locally into the region of interest rather than systemically through intravenous, intraperitoneal, or subcutaneous injections. For brain imaging, that means that intrathecal and intracerebral injections will have to be used. Such injections are technically challenging and hardly (if anyhow) translatable to the clinic. Therefore, this paradigm has not yet been used for head‐to‐head comparisons of agonist and antagonist radioligands.

### 5.3 Current evidence on the existence of high‐affinity state in vivo

#### 5.3.1 Dopamine receptors

The majority of studies attempting to demonstrate the existence of high‐affinity state in vivo has concerned dopamine D_2/3_ receptors. Agonists [^11^C](−)NPA, [^11^C]MNPA, and [^11^C](+)PHNO were compared with antagonists [^11^C]raclopride and [^18^F]fallypride. In some studies, these compounds were used in their unlabeled or ^3^H‐labeled forms. A large portion of these studies failed to demonstrate the existence in vivo of the subpopulation of D_2/3_ receptors configured in the high‐affinity state (see Skinbjerg et al^215^ for review).

Binding site densities for D_2/3_ agonist and antagonist tracers were found to be equal in one study,^90^ while in another study the average relative abundance of D_2/3‐high_ was found to be 79%,^193^ which is close to the upper extreme of such percentages determined in vitro.^216^ Saturation of [^11^C](+)PHNO binding in monkey brain was found to be biphasic but the two binding sites most probably corresponded to D_2_ and D_3_ receptor subtypes rather than to high‐ and low‐affinity states.^217^

In rats with brain lesions induced by the dopaminergic neurotoxin 6‐hydroxydopamine, binding levels of D_2/3_ antagonist [^11^C]raclopride and of D_2/3_ agonist [^3^H]PHNO were increased to the same extent.^194^ No difference in baseline agonist binding relative to the healthy condition was found in dopamine β‐hydroxylase knockout (Dβh‐KO) mice,^103^ in rats withdrawn from chronic ethanol and in amphetamine‐sensitized rats.^194^ In these three animal models, an upregulation of D_2/3‐high_ was previously demonstrated in vitro by the group of Seeman et al.^201^, ^218^, ^219^, ^220^ However, in vitro measurements of elevated striatal D_2/3‐high_ in Dβh‐KO mice could not be replicated by the group that performed the in vivo imaging study.^103^ Moreover, most of the data on the elevation of D_2/3‐high_ in ethanol‐withdrawn rats is based on comparison of *B*_max_ values for antagonist radioligands in the presence and absence of GppNHp,^201^, ^219^ an indirect method of assessing D_2/3‐high_ abundance, shortcomings of which are discussed in Van Wieringen et al.^216^

In clinical studies of diseases where alteration of D_2/3‐high_ was suspected, binding potentials of agonist tracers in healthy and diseased subjects were similar,^202^, ^221^, ^222^, ^223^, ^224^ although some recent reports buck this trend.^195^, ^225^

The dopamine D_2/3_ antagonist radioligand raclopride (^11^C‐ or ^3^H‐labeled) was displaced by D_2/3_ agonist drugs in a monophasic manner.^86^, ^196^, ^197^ In a more recent study, up to 70% of [^11^C]raclopride binding was displaced by D_2/3_ agonist quinpirole without any evidence of biphasicity.^39^

The majority of studies comparing agonist and antagonist tracers’ vulnerability to displacement by agonist drugs found no difference in vulnerabilities,^86^, ^107^, ^196^ though some reports confirming greater vulnerability of agonist tracers do exist 199, 200 and the relative timing of tracer and drug administration were claimed to be important.^200^

Nevertheless, D_2/3_ agonists did prove to be more sensitive than antagonists to endogenous dopamine levels in anesthetized rodents,^44^ cats,^90^ primates,^92^, ^98^, ^106^ as well as in awake humans,^93^, ^102^ though not in awake rodents,^86^, ^194^, ^196^, ^203^ with a single exception.^199^ However, lack of consistency in preclinical data on neurotransmitter and agonist drug challenge raises a question whether this advantage of the agonists stems from their preference for the high‐affinity state or from other factors (see Section 5.2.2).

For dopamine D_1/5_ receptors, an in vivo displacement curve was built in baboons using the D_1_‐antagonist [^11^C]NNC‐112 and the D_1_‐agonist drug DAR‐0100A.^198^ Occupancies above 40% were not investigated, but the best‐fit curve was monophasic, not supporting the existence of a high‐affinity receptor subpopulation.

#### 5.3.2 Serotonin receptors

A few head‐to‐head agonist‐antagonist comparisons done with serotonin receptor radioligands yielded ambiguous results.

Two studies found that about 80‐90% of the specific binding of antagonist 5‐HT_1A_ ligand [^11^C]WAY‐100635 could be displaced by the 5‐HT_1A_ agonist 8‐OH‐DPAT,^226^, ^227^ but in both cases only a single dose of the agonist was tried, so it is impossible to say whether 8‐OH‐DPAT displaces [^11^C]WAY‐100635 in a monophasic or a biphasic manner. In an ex vivo saturation study, the binding site density of 5‐HT_1A_ agonist [^3^H]CUMI‐101 was 33% lower than that of the antagonist [^3^H]MPPF in rat frontal cortex, but 82% higher in rat hippocampus.^122^ BP_F_ values for [^11^C]CUMI‐101 across the baboon brain were about 45% of BP_F_ values for 5‐HT_1A_ antagonist [^11^C]WAY‐100635,^70^ while in vitro *K*_d_ values for the two tracers were comparable, but these findings should be interpreted with caution for reasons described in the subsection “Extracting density values from true binding potential measurements” of Section 5.2.1. Yet another study found considerable differences between the binding patterns of 5‐HT_1A_ agonist [^18^F]F13174 and 5‐HT_1A_ antagonist [^18^F]MPPF in marmosets: regional BP_ND_ values even did not correlate when animals were imaged without the use of anesthesia.^132^ However, the variability of data (especially for [^18^F]F13174) was high, so it is hard to draw any firm conclusions regarding the existence of separate 5‐HT_1A_ receptor subpopulations recognized by [^18^F]F13174 and [^18^F]MPPF from the study.

Regional BP_ND_ values of the 5‐HT_2A_ agonist [^11^C]CIMBI‐36 were compared with regional BP_ND_ values of the 5‐HT_2A_ antagonist [^11^C]MDL100907 in monkeys 137 and regional BP_ND_ values of another 5‐HT_2A_ antagonist [^18^F]altanserin in humans.^139^ Both analyses did yield deviations from linear correlation in the hippocampus and choroid plexus, but these could all be explained by the binding of [^11^C]CIMBI‐36 to 5‐HT_2C_ receptors. In a recent non‐human primate study, [^11^C]CIMBI‐36 turned out to be more sensitive than [^11^C]MDL100907 to fenfluramine‐induced serotonin release,^142^ but, as discussed above for D_2/3_ radioligands, this does not per se prove that [^11^C]CIMBI‐36 preferentially binds to the high‐affinity state of 5‐HT_2A_ receptors in vivo.

To sum up, the majority of data, both for dopamine and serotonin receptors, does not directly support the existence of a receptor subpopulation in vivo to which agonists preferentially bind. There are some undisputable differences in the behavior of agonist tracers and antagonist tracers, such as specific sensitivity of the former to anesthesia and greater sensitivity to synaptic neurotransmitter levels, but the reason for these differences is not clear.

### 5.4 Experimental data in light of the nature of the high‐affinity state

Some attempts to detect the high‐affinity state in vivo may have failed because the used radioligands were not sufficiently subtype‐selective or lacked sufficient intrinsic acitivity via the canonical GPCR pathway.^118^, ^119^, ^228^ In other cases, however, the reasons for failure were completely unclear. Therefore, the nature and functioning of the high‐affinity state in vivo has remained elusive.

Two explanations of the failure to detect high‐ and low‐affinity states in vivo have been put forward. One explanation proposes that all, or almost all, receptors are configured in the high‐affinity state in vivo 86 and permanently precoupled to G‐proteins, so that these G‐proteins, at least their Gα subunits, do not dissociate from the receptors after activation.^215^ Another explanation states that, whatever the baseline degree of receptor‐G‐protein precoupling, receptors can and do recruit new G‐proteins when occupied by agonists, so high agonist concentrations eventually make all receptors bind G‐proteins and thus convert to the high‐affinity state.^196^, ^198^ Indeed, receptors have been subjected to high agonist (drug or neurotransmitter) concentrations in virtually all experimental paradigms used for in vivo detection of the high‐affinity state **(**Table 2 **)**. In vivo imaging experiments last for tens of minutes, while time constants for receptor and G‐protein activation and for receptor‐ligand and receptor‐G‐protein binding vary from tens of milliseconds to a few seconds.^37^ Gradual G‐protein recruitment in response to agonist binding can thus confound experimental outcomes in currently used paradigms and can “inflate” the apparent relative abundance of the high‐affinity state in vivo. Indeed, in studies comparing the vulnerability of dopamine D_2/3_ agonist and antagonist tracers to drug challenge, the lowest doses of D_2/3_ agonist drugs and the dopamine release stimulator amphetamine (resulting in the lowest receptor occupancies and therefore minimal G‐protein recruitment) tended to produce the greatest relative difference between agonist and antagonist tracer displacement. In three such studies, the lowest dose of challenge drugs resulted in zero or negative displacement of the antagonist [^11^C]/[^3^H]raclopride but positive (though small and not statistically significant) displacement of agonist tracers [^11^C]/[^3^H]PHNO and [^11^C]/[^3^H]MNPA.^86^, ^107^, ^196^ Superior sensitivity of D_2/3_ agonists to amphetamine‐induced dopamine release in humans compared to the D_2/3_ antagonist [^11^C]raclopride was demonstrated at receptor occupancies of 22% or lower.^93^, ^102^ Finally, the D_2/3_ agonist [^3^H]PHNO was found to be more vulnerable than [^3^H]raclopride to displacement by the agonist (−)NPA when this agonist was co‐injected with the radioligands instead of being injected sometime before their administration, although this study used non‐normalized “raw” specific binding as outcome measure.^200^

Comparison of the in vivo imaging results with available in vitro data on detection of the high‐affinity state raises even more questions. There are no systematic reviews of in vitro studies assessing the percentage of receptors configured in the high‐affinity state for nondopaminergic receptors. However, recently published reviews dedicated to the affinity states of D_2/3_ receptors 216, 229 demonstrate not only the lack of agreement but also the absence of a trend in the assessments of the abundance of the high‐affinity state of D_2/3_ receptors in different setups**:**

(i) Experiments in membrane homogenates from transfected cells and isolated tissues consistently showed that a significant portion of D_2/3_ receptors is configured in the low‐affinity state. Only about 20% of dopamine D_2/3_ receptors in the rodent brain and about 30% in the human brain are in the high‐affinity state.^216^
(ii) No high‐affinity state of D_2/3_ receptors could be detected on intact cells. In bovine pituitary membranes, 55% of D_2/3_ receptors were in the high‐affinity state, but intact dispersed pituitary cells had only low‐affinity state.^230^ Similar results were demonstrated with transfected HEK293T and T‐REx‐293 cells expressing D_2_ receptors.^28^, ^42^ One study found 16% of D_2_ receptors on cultured rat pituitary adenoma cells configured in the high‐affinity state using [^3^H]domperidone as a radioligand,^231^ but these results have yet to be replicated.
(iii) Autoradiographic studies where densities (B_max_) of D_2/3_ receptors in tissue slices were estimated, tended to produce lower B_max_ values for agonist than for antagonist radioligands, but the variance between studies was too high to consider the difference significant.^229^ The majority of antagonist vs agonist drug displacement experiments demonstrated a single high‐affinity population of D_2/3_ receptors in tissue slices in vitro.^229^ This is in agreement with the data from in vivo imaging (see Section 5.3.1).

To explain the low relative abundance of the high‐affinity state in membrane homogenates within the precoupling model of receptor‐G‐protein interaction, one can assume that partial dissociation of receptor‐G‐protein complexes occurs during membrane preparation, but the exact mechanism is hard to define. Alternatively, one can assume the existence of a large intracellular reserve of receptors in the high‐affinity state. In transfected cells, the affinity of agonist ligands used as PET tracers toward internalized D_2/3_ receptors was shown to be about twofold lower than toward surface receptors,^42^ a change that would hardly be noticeable in saturation or competition curves. However, the total densities of D_2/3_ receptors measured in membrane homogenates by radioligand binding saturation and in vivo by PET are in good agreement,^229^ which does not support the existence of extra D_2/3_ receptors that are detectable in vivo but are not found in membrane homogenates.

The collision coupling model of receptor‐G‐protein interaction can be reconciled with measurements of the high‐affinity state in membrane homogenates if one assumes that in these homogenates only a fraction of the total G‐protein pool of the cell is available for recruitment to the receptors. Indeed, the number of G‐proteins in living cells is likely equal to or much greater than the number of their cognate receptors.^45^, ^232^, ^233^ However, given that G‐proteins are anchored to the lipid bilayer,^234^ it is not clear how such G‐protein reserve can both be easily accessible in the living tissue and become lost in membrane homogenates. Especially the findings of Seeman^200^ are puzzling in this regard. In that study, IC_50_ values for the displacement of [^3^H](+)PHNO and [^3^H]raclopride from striatal membranes by (−)NPA were measured. When radioligands and (−)NPA were added to the membranes simultaneously, the IC_50_ value observed for [^3^H](+)PHNO was sevenfold lower than for [^3^H]raclopride. However, when (−)NPA was added to the membranes 30 minutes earlier than the radioligands, the difference in IC_50_ values for [^3^H](+)PHNO and [^3^H]raclopride was only twofold. Similar vulnerabilities of [^3^H]raclopride and [^3^H](+)PHNO in the case of (−) NPA pre‐incubation could mean that (−)NPA can and does stimulate G‐protein recruitment to all or almost all receptors in the membranes, while greater vulnerability of [^3^H](+)PHNO in case of simultaneous addition may mean that in the absence of an agonist, only some receptors are G‐protein‐bound and therefore accessible to [^3^H](+)PHNO. However, in those experiments, radioligands were equilibrated with membrane homogenates for 2 hours before readout, which is much longer than the pre‐incubation step. Therefore, even in the case of simultaneous addition of radioligands and (−)NPA, there should have been enough time during the equilibration step for (−)NPA to elicit recruitment of “spare” G‐proteins to the receptors.

The absence of the high‐affinity state of D_2/3_ receptors in isolated intact cells ^28,42,230^ is even harder to reconcile with either of the two models mentioned above. Collision coupling at least provides a theoretical explanation of the disappearance of high‐affinity state upon agonist addition due to a high level of GTP in living cells. Still it remains puzzling why agonists force D_2/3_ receptors in dispersed cells from a natural tissue (bovine pituitary) to uncouple from G‐proteins (convert into the low‐affinity state), but promote G‐protein recruitment (conversion to the high‐affinity state) when they are acting on the same receptors in intact tissue.

Switching from the G‐protein‐dependent high‐affinity state model to the oligomerization‐dependent model leaves the same questions open: it is not clear how the degrees of receptor oligomerization can be different in membranes, dispersed cells and living tissues. Moreover, observation of almost all receptors configured in high‐affinity state in vivo is hard to reconcile with the oligomerization‐dependent model. At full oligomerization and full agonist occupancy, cooperativity‐induced high‐affinity state can have a relative abundance of no more than 50% (in the case of dimers), and higher values imply that few, if any, receptors are oligomerized.

## 6 CONCLUSION

The concept of the high‐affinity state postulates that a certain subset of receptors in the living brain is primarily responsible for signaling. Assessing the abundance of this subset is thus potentially very relevant for studies concerning the responses of neurotransmission to pharmacological or physical stimuli and the dysregulation of neurotransmission in neurological disorders.

A number of experimental paradigms have been developed for the estimation of the relative abundance of receptors configured in the high‐affinity state. The high‐affinity state is preferentially recognized by agonists in vitro, so the development of agonist PET tracers as tools for the noninvasive imaging of the high‐affinity state has become popular in recent decades.

The greatest number of agonist tracers has been developed for dopamine D_2/3_ receptors, but agonist tracers for dopamine D_1_, µ‐opioid, and muscarinic M_2_ receptors are also known, and in recent years, radiolabeled agonists for serotonin 5‐HT_1A_ and 5‐HT_2A_, κ‐opioid and muscarinic M_1_ receptors have appeared. It should be noted, however, that for many of the nondopaminergic tracers the actual preference for the high‐affinity state has not been directly tested, because functional agonism is often assumed to imply preferential binding to the high‐affinity state.

For dopamine, serotonin and κ‐opioid receptors, head‐to‐head comparisons of agonist and antagonist tracers are now possible, while matching antagonist tracers for muscarinic M_2_ and µ‐opioid receptors have yet to be developed. Given that, beyond head‐to‐head agonist‐antagonist comparisons, antagonist tracers are also suitable for experiments like displacement curve generation (see Section 5.2.2), development of new antagonist tracers for muscarinic M_2_ and µ‐opioid receptors with a pharmacological selectivity matching that of existing agonist tracers will arguably be more useful for the assessment of the affinity states of these receptors than development of new agonist tracers.

Agonist tracers appear to be more sensitive to endogenous neurotransmitter challenge, as was originally expected. However, other expectations regarding agonist tracers have not been fulfilled. Agonist imaging did not reveal alterations in the relative abundance of the high‐affinity state in neurological disorders. The benefits of agonist tracers for the imaging of receptor occupancies by drugs have also not been proven.

Moreover, though the separation of GPCRs into subsets with high‐ and low‐affinity state is consistently observed in membrane homogenates in vitro, data from preclinical and clinical experiments do not support the existence of the high‐ and low‐affinity states in vivo. The majority of these data concerns dopamine D_2/3_ receptors but recent results on serotonin receptors paint the same picture.

The relative abundance of the high‐affinity state in vivo may simply be close to (or equal to) 100%, making the detection of low‐affinity state unfeasible. It is also possible that agonist drugs or tracers used for in vivo experiments may inflate the relative abundance of the high‐affinity state.

Critical revision of experimental approaches and collection of experimental evidence for nondopaminergic receptors will help clarify whether the high‐affinity state of GPCRs exists in vivo and whether agonist tracers really have advantages over antagonist tracers because of their preferential binding to the high‐affinity state.

## Figures and Tables

**Figure A1:**
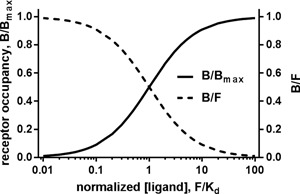
Relationship between the fraction of ligand‐occupied receptors and *B*/*F* ratio. At equilibrium, *B*/*B*_max_ = *F*/(*F* + *K*_d_), therefore *B*/*F* = *B*_max_/(*F* + *K*_d_). If *F* ≪ *K*_d_, then *B* ≪ *B*_max_ and *B*/*F* ~ *B*_max_/*K*_d_. Note that at low concentrations (*F*/*K*_d_ ≪ 1), the ligand occupies only a small part of total receptor population, and the *B*/*F* ratio (binding potential) is stable (left part of the graph)

## APPENDIX A

### CALCULATION OF BINDING POTENTIALS

The ratio of the concentrations of specifically bound tracer (*B*) and free tracer (*F*) in the tissue at equilibrium is equal to the ratio of available receptor density and ligand‐receptor affinity (*B*_max_/*K*_d_) when the tracer only occupies a negligible fraction of all receptors (Figure A1).

In practice, specifically bound tracer concentration *B* is often calculated as a difference between tracer concentrations in a receptor‐rich region of interest and a receptor‐poor reference region. Free tracer concentration, on the other hand, is replaced with directly measurable concentrations that are linearly dependent on “true” *F* at equilibrium.^192^ These concentrations can be:

– Free tracer concentration in plasma (equal to *F* if the tracer is transferred from tissue to plasma by passive diffusion);
– Total, that is, free and protein‐bound, tracer concentration in plasma;
– Nondisplaceable, that is, free and nonspecifically bound, tracer concentration in tissue.

To determine tracer concentration in plasma (whether free or total) one needs to obtain arterial plasma samples, which is an invasive procedure. Therefore, the last option is the most popular: tracer concentration in receptor‐rich tissue is related to tracer concentration in receptor‐poor tissue.

Binding potentials are defined at equilibrium. Plasma and tissue concentrations of a tracer can be directly equilibrated with each other by administering the tracer in an infusion but such paradigms are hard to implement. Therefore, in most positron emission tomography (PET) and single‐photon emission computed tomography (SPECT) studies tracer is administered via a simple bolus injection. In that case, to calculate binding potentials, time‐activity curves for regions of interest are used as input data for so‐called kinetic modeling. A compartmental model is built up based on relevant theories. Tracer distribution is described in terms of its transfer between virtual compartments (like “free tracer,” “nonspecifically bound tracer,” and “specifically bound tracer”). The model is fitted to the time‐activity curves acquired by the PET camera. If the model can adequately describe the experimental curves, the values of individual kinetic parameters or their combinations are determined from the model fit. Fewer fitting parameters often mean a more robust fit, so many simplifying assumptions are often made. Graphical analyses, simplifying the fitting process to linear regression, are also widely used.^235^, ^236^ Fitted parameters are then used to obtain binding potentials, essentially by extrapolating the kinetics observed in the imaging experiments to the situation of equilibrium.
